# Editorial: The role of neuroimaging and neurostimulation in detecting and treating Alzheimer's disease and mild cognitive impairment

**DOI:** 10.3389/fnhum.2024.1524921

**Published:** 2024-11-25

**Authors:** Arianna Menardi, Rachel A. Crockett

**Affiliations:** ^1^Department of Neuroscience, University of Padova, Padova, Italy; ^2^Padova Neuroscience Center, University of Padova, Padova, Italy; ^3^Nuffield Department of Clinical Neurosciences, University of Oxford, Oxford, United Kingdom

**Keywords:** Alzheimer's disease, neuroimaging, neuromodulation, magnetic resonance imaging, mild cognitive impairment, transcranial magnetic stimulation

## Background

Alzheimer's disease (AD) is the most common cause of dementia, characterized by progressive accumulation of amyloid-β plaques and tau protein neurofibrillary tangles, functional and structural dysconnectivity, and widespread cognitive impairment (Korczyn and Grinberg, 2024). Due to the multifactorial nature of this disorder, no effective individual pharmaceutical treatments have yet been identified (Korczyn and Grinberg, 2024). Thus, research has become increasingly focused on identifying early biomarkers of pathology that can predict disease progression and guide interventions to preserve brain health. Mild cognitive impairment (MCI) is the prodromal stage of AD and is characterized by cognitive impairment beyond what is expected for one's age or level of education (Nasreddine et al., 2005). Not all individuals who experience MCI symptoms will progress to AD (Farias et al., 2009), making it a promising window for intervention to prevent disease progression. Critically, the heterogeneity of MCI conditions has limited the ability to pinpoint a single conversion mechanism. Using advancements in technology, it is possible to incorporate multi-factorial models that can provide a more holistic evaluation of this multifaceted condition.

Success in identifying early biomarkers of pathology and modeling the disease progression are two building blocks necessary for the development of successful interventional therapies. Non-pharmacological interventions to enhance residual cognitive function, such as non-invasive neurostimulation, have grown in popularity due to their limited side effects and flexibility in adapting protocols for individualized therapy. However, the optimal targets and stimulation parameters needed to elicit longer-term cognitive benefits have yet to be identified.

## Early structural biomarkers of pathology

Cortical atrophy is a hallmark of aging, characterized by volume loss at the whole-brain level (Raji et al., 2009). In older individuals developing AD pathology, such atrophic patterns appear exacerbated in hippocampal and entorhinal cortex regions (Peng et al., 2015; Raji et al., 2009), resulting in cognitive deficits that eventually require clinical attention, i.e., episodic memory failure. More recently, the call for the identification of early biomarkers of AD has guided investigations toward identifying patterns of atrophy that occur well before a formal diagnosis. In this regard, Hu et al. investigated the discriminative power between volumetric loss due to normal aging and AD-specific pathology. Using retrospective longitudinal analysis, they identified that greater levels of atrophy in the frontal and temporal lobes preceded the onset of AD, which was exacerbated in EPOE ε4 mutation carriers.

However, volumetric alterations do not happen in isolation but are often accompanied by cerebrovascular changes, such as white matter hyperintensities (WMH; Hu et al., 2021). Given the tight link between vascular pathology and neurodegeneration, Cao et al. identified that greater WMH volumes were associated with greater atrophy of specific brain regions within the temporal lobe and insula. The relationship between vascular lesions, atrophy, and neurodegeneration lends support for the need to promote lifestyle interventions known to modify these risk factors (e.g., obesity, hypertension, diabetes, smoking, and physical inactivity) (Livingston et al., 2017).

## Modeling AD pathology

Given the multifactorial nature of AD, models should consider clinical, biological, molecular, genetic, and neuropsychological variables in combination. By combining information from cognitive tests, cortical thickness, and amyloid-β positron emission tomography (PET) scans, Jung et al. developed a deep learning model with high predictability of cognitive and structural alterations over time in MCI patients. Jiang et al. also evaluated the performance of several machine learning models in discriminating between cognitively normal and AD participants. Interestingly, in their approach, the authors used cortical complexity, described as the fractal dimension of the cortical surface, as a more sensitive measure of atrophy than traditional volumetric analyses (Nicastro et al., 2020). Overall, the use of machine learning is becoming increasingly popular given the ability of such models to deal with the high-dimensional, non-linear data necessary to output individual-centered predictions.

Animal models provide an alternative approach to investigating the pathological mechanisms of AD. Indeed, the shorter lifespan (e.g., in rat models) allows to monitor the evolution of the disease phenotype in a relatively short period, with the possibility to directly exert control over the experimental variables. De Waegenaere et al. identified changes in brain states between the pre-and early-plaque stages in rat models that involved networks equivalent to the default mode network, lateral cortical network, and basal forebrain regions in humans.

Both machine learning and animal models provide important opportunities to determine the pathological features of AD and can help us characterize the early impact and biomarkers of disease progression.

## Non-invasive neuromodulation interventions

In this Research Topic, Xu et al. reported on the beneficial effects of Moxibustion treatment for increased functional connectivity and cognitive functioning, whereas Kim et al. presented a study protocol for the implementation of a personalized intervention using transcranial direct current stimulation. The recent evolution in the field arises from the necessity to overcome the limitations of “traditional symptom- and sign-based diagnoses and clinical one-size-fits-all interventions,” which have long failed in heterogeneous diseases such as AD (Hampel et al., 2019). However, the vast array of neuromodulation techniques, sites of stimulation, and criteria for inclusion require greater refinement. Among the possible ameliorative interventions, there is a need for personalized protocols based on individual head models (Menardi et al., 2022). Information on the individual neuroanatomy can be used to predict the amount of electrical current reaching the target area and further implement search algorithms for the optimization of electrode/coil locations on the scalp (Gomez et al., 2021; Miranda et al., 2018).

## Conclusion

AD represents a complex and multifactorial disease for which no cure is available. Precision medicine approaches call for individually tailored therapeutic modalities based on the multimodal integration of patient data to establish preventive, predictive, personalized, and participatory interventions (Hampel et al., 2023). Current efforts revolve around: (i) defining early biomarkers of pathology to identify individuals at risk, (ii) modeling the disease course to predict pathological progression, and (iii) defining patient-centered therapies to increase resilience and help counteract pathological progression.
